# Mesenchymal/stromal stem cells: necessary factors in tumour progression

**DOI:** 10.1038/s41420-022-01107-0

**Published:** 2022-07-22

**Authors:** Xinyu Li, Qing Fan, Xueqiang Peng, Shuo Yang, Shibo Wei, Jingang Liu, Liang Yang, Hangyu Li

**Affiliations:** Department of General Surgery, The Fourth Affiliated Hospital, China Medical University, Shenyang, 110032 China

**Keywords:** Cancer, Molecular biology

## Abstract

Mesenchymal/stromal stem cells (MSCs) are a crucial component of the tumour microenvironment (TME). They can be recruited from normal tissues into the TME and educated by tumour cells to transform into tumour-associated MSCs, which are oncogenic cells that promote tumour development and progression by impacting or transforming into various kinds of cells, such as immune cells and endothelial cells. Targeting MSCs in the TME is a novel strategy to prevent malignant processes. Exosomes, as communicators, carry various RNAs and proteins and thus link MSCs and the TME, which provides options for improving outcomes and developing targeted treatment.

## Facts

1. MSCs in the TME can be recruited from other tissues, and many MSCs are located in the TME (some MSCs are called CAFs (cancer-associated fibroblasts)); the relevant recruitment mechanisms are a hot spot of research.
2. MSCs have stemness and can transform into other cell types, which is a key characteristic for tumour cell survival.
3. This stemness of MSCs may be passed to tumour cells and induce CSCs to proliferate.
4. Exosomes from MSCs carry various factors that aid cell-to-cell communication, and therefore, exosomes may be a target for preventing tumour progression.

## Open questions

1. MSCs how to move to the location in the metastasis niche and primary microenvironment?
2. How many cell types of MSC can converted into and what function of these cell types in tumour progression?
3. Whether MSC exosomes can be a meaningful tool for future therapy?

## Introduction

In the 1960s, a subtype of fibroblasts originating from bone marrow that possessed colony forming capacity and existed in almost all tissues was identified [1, 2]. These “fibroblasts” with the capacity to self-renew and differentiate were called mesenchymal stem cells (also named mesenchymal stomal cells) (MSCs) [3–5]. MSCs are a heterogeneous population that possess fibroblast-like morphology and the capacity to form colonies, and they proliferate in vitro as plastic-adherent cells [6]. MSCs can be isolated from different tissues, and their functions are distinct. In this review, we mainly focus on MSCs in tumours.

There is a general consensus that human MSCs do not express haematopoietic markers, such as CD14, CD34, CD40, CD45, CD80, and CD86, but they do express some markers, such as CD44, CD71, CD73, CD90 (THY1), CD105 (endoglin), and CD271 (low-affinity nerve growth factor receptor) [2]. The expression patterns of these markers are variable and probably depend on tissue source, species and culture conditions.

The International Society for Cryotherapy (ISCT) suggests three features for defining MSCs (in culture) [7]: (i) plastic adherence of the isolated cells in culture; (ii) CD105, CD73, and CD90 expression in greater than 95% of cultured cells and lack of CD34, CD45, CD14 or CD11b, and CD79a expression or CD19 and HLA-DR expression in greater than 95% of cultured cells; and (iii) capacity to differentiate into bone, fat and cartilage (Table 1).

**Table 1:** BM-MSC markers.

PMID	CD14	CD20	CD29	CD34	CD45	CD73	CD90	CD105	CD106	CD146	CD235	CD271	HLA-DR
31123087	−	−	/	−	−	**+**	**+**	**+**	**/**	**/**	**/**	**/**	−
23656504	−	/	/	−	−	**+**	**+**	**+**	/	/	/	/	−
16923606	−	/	/	−	−	**+**	**+**	**+**	/	/	/	/	−
25966666	/	/	/	−	−	**+**	**+**	**+**	/	/	/	/	/
21424106	/	/	/	−	−	/	**+**	/	/	/	/	/	/
31844519	−	/	**+**	−	/	**+**	/	/	/	/	/	/	/
32868394	/	/	/	−	−	**+**	/	**+**	/	/	/	/	/
					−								

The MSC criteria of the ISCT have not been optimized because of the complexity and heterogeneity of MSCs [8]. For example, cells that are freshly isolated may show different cell surface molecules than those that have been cultured. In melanoma, CD146 and CD271 have both been described as markers of freshly isolated MSCs, but in cells that have been grown in culture media containing bFGF, CD271 expression is lost [9]. Similarly, when cells are cultured in media, CD146 expression is upregulated, and under hypoxic conditions, it is downregulated [10]. Additionally, in MSCs derived from mice or humans, CD44 marks MSCs that have been cultured in media, but uncultured MSCs do not express CD44 [11]. Recent studies have indicated that the characteristics of MSCs are plastic, and MSCs may express different cell surface markers according to culture conditions. Importantly, MSCs cultured in media do not represent MSCs in vivo. A study of MSCs noted that they have trilineage differentiation capacities in vitro but exhibit limited differentiation capacities in vivo [12]. Consequently, using specific factors to classify and identify MSCs in unique conditions may be a novel and promising strategy, and to test this, we collected cell marker data based on culture conditions in vivo and in vitro.

## MSC recruitment to tumours

MSCs exist in almost all types of tissues regardless of physiology and pathology; normal physiological conditions maintain tissue homoeostasis and repair, and pathological conditions are involved in disease progression [13–15]. The links between MSCs and infection, chronic inflammation, and tumour progression have long been recognized [16, 17]. Not only can MSCs be recruited to tumours but MSCs (but not HSCs) can acquire a gastric mucosal cell gene expression pattern without cell-to-cell contact or fusion in gastric tumours (related to H. felis infection) [18]. This suggests that epithelial tumours originate from bone marrow MSCs. Under chronic inflammation, MSCs are recruited to inflammation sites and can transform in the TME; in addition, they can also lead to tumour growth and metastasis. Consequently, the tumour tropism of MSCs is an important factor involved in tumorigenesis and development.

MSCs have been assessed various studies, and one such study monitored the dynamic activity of cells by assessing specific luciferase activity in mice. Movement of MSCs to tumours was observed regardless of whether the MSCs were administered via intravenous or intraperitoneal injection [19]. In one study, CD105-positive cells were found in pulmonary arterial blood in greater than 90% of lung cancer patients [20]. Similarly, in a study of human gastric tumours, MSCs were present in 13 out of 20 tissue samples [21]. A report based on the fluorescence labelling of endogenous MSCs verified that cells can migrate into tumours [22, 23]. However, the abovementioned investigations employed xenotransplantation of immunodeficient mice to explore tumorigenesis and tumour progression. Although some deficiencies exist, these models are extremely helpful for comprehending the relationship between MSCs and the TME, especially in the host adaptive immune system. Intriguingly, L-MSCs (lymphoma-derived MSCs) induce stronger direction of monocytes/macrophages than BM-MSCs (bone-marrow-derived MSCs), and this activation may be increased by CCR2 ligands, which promote tumour growth in vivo [23]. A hypothesis by Paget in 1889 proposed an irreplaceable role of the tumour microenvironment in survival and metastasis, which clarified the bidirectional communication between the “seed” (tumour cells) and “soil” (the microenvironment) [24]. MSCs can be recruited to the tumour microenvironment (TME) and acquire new features as a result of continuous exposure to local factors, leading to the overexpression of CCR2 ligands that recruit monocytes/macrophages and neutrophils [23]. Notably, in glioma, the mouse models with fibroblasts showed similar results that BM-MSCs tumour tropism in vivo [25]. In human breast tumours, MSCs migrated much faster (11-fold faster) towards CM from tumour cells than toward control CM, and as assessed by an intravenous technique in vivo, green fluorescent protein (GFP)-labelled human MSCs were found to localize in developing tumours but showed no obvious accumulation in other tissues in mice [26]. In a TEB (tissue-engineered bone) model with fluorescently labelled human BM-MSCs, confocal imaging and fluorescence-activated cell sorting (FACS) indicated that MSCs can home to the tumour after 2.5 weeks [27]. GFP-MSCs (100000 cells) derived from mice were found near the tumour and accounted for 0.05%–0.1% of all cells [28]. This evidence clarified that the tumour tropism of MSCs is mediated in different ways even in TEB models, and this tropism also exists in various tumours.

CXC chemokine ligand 16 (CXCL16) is the ligand for CXCR6. CXCR6 is a receptor of the seven-transmembrane G protein-coupled receptor family that is mainly expressed on the surface of lymphocytes (CD4^+^ T cells, CD8^+^ T cells, NKT cells, and NK cells). A growing number of reports have noted the role of CXCL16-CXCR6 signalling in mediating the chemotaxis of multiple cells [29, 30]. Microarrays of tissues from prostate cancer patients showed high expression of CXCL16 in tumour compared with adjacent normal tissues, and the expression of CXCL16 was related to tumour growth. In tumour tissues, there were more MSCs in CXCR6^+/+^ mice than CXCR6^−/−^ mice, suggesting specific recruitment of MSCs into tumours via the CXCL16-CXCR6 axis [31]. In addition, one of the most serious complications of prostate tumours is skeletal metastases, and the CXCL16/CXCR6 axis functions in chemoattraction to induce metastasis [32]. In summary, CXCL16 and its receptor play an important role in the tumour tropism of MSCs, but the biological mechanisms by which cell movement is triggered and supporting the production of energy for the MSC migration process remain to be further studied.

**Fig. 1:**
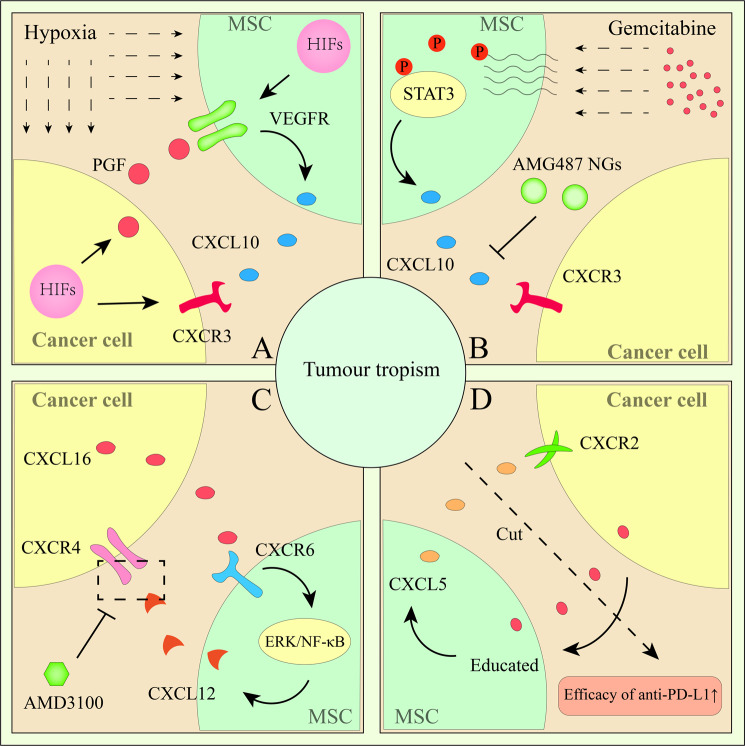
Mechanism by which tumour cells recruit MSCs into the TME. **A** Hypoxia can induce HIF expression and then promote the release of PGF and CXCL10, leading to the recruitment of MSCs into the TME. **B** Gemcitabine activates STAT3 phosphorylation in MSCs to promote CXCL10 release, which mediates the MSC recruitment process. AMG487 can prevent this process to improve gemcitabine efficacy. **C** Tumour cell-related CXCL16 activates CXCR6/ERK/NF-kB signalling, leading to CXCL12/CXCR4 activation in MSCs, and CXCL12/CXCR4 promotes the recruitment of MSCs into the TME. AMD3100 prevents this process. **D** Tumour-educated MSCs induce CXCL5 release to activate CXCR2 signalling in tumour cells, which promotes the tumour tropism of MSCs. The efficacy of anti-PD-L1 drugs can be enhanced by restraining the communication between MSCs and tumour cells.

In addition to the above factors, other factors may also be involved in migration and homing. In vitro, CM (conditioned medium) from breast tumour cells cultured under hypoxic conditions induced increased homing by HIF (hypoxia-inducible factor)/PGF (placental growth factor) and VEGFR1 signals compared with normal conditioned medium [33]. Chemotherapy can educate MSCs, causing more MSCs to be recruited to tumours, and these educated MSCs were found close to TICs (tumour-initiating cells) in pancreatic tumours because of processes is mediated by CXCL10-CXCR3 signalling; AMG487-loaded nanoghosts can interrupt this process [28]. TME stress and inflammation may promote MSC-tumour tropism. In different tumours, receptors of chemokines and cytokines on cells have their own expression patterns, which explains the important differences in the functions of factors in different tumours (Fig. 1).

## Effect of MSCs on tumours

According to previous studies, MSCs have vital functions in tissue regeneration, cellular homoeostasis and microenvironment homoeostasis and are involved in many processes, including inflammation, cellular proliferation, cellular migration and immune processes [2, 3, 34]. In the process of wound healing, inflammatory mediators drive the migration of MSCs, leading to the proliferation and differentiation of MSCs [35, 36]. That MSCs function in wound healing proved that they can actively integrate into damaged tissues to mediate the tissue repair process. Notably, MSCs extensively participate in the regulatory process of the microenvironment in normal and pathology states. MSCs not only promote wound healing but can also alter the microenvironment according to the unique pathophysiological status of the tissues in which they reside [35, 36]. Tumours induce microenvironment changes. The pathological state of tumours can cause migration of MSCs, which then evolve into cells such as tumour-associated MSCs (TA­MSCs: MSCs residing in the TME including TA-MSCs and tumour-educated MSCs) and cancer-associated fibroblasts (CAFs: activated fibroblasts (also referred to as myofibroblasts) that are found in association with cancer cells) [22, 23, 37]. In addition, phenotypical and functional heterogeneity in tumours is crucial for tumour characteristics, making it difficult for treatments to eradicate all tumour cells for many solid tumours and usually leading to tumour recurrence [38]. Heterogeneity is mainly caused by genomic instability of the tumour-initiating cells and stressors (such as acidosis, hypoxia, immune responses, and drugs) in the TME [39, 40]. The recent identification of stem cells in various tumour tissues has attracted the interest of researchers, and recently, stem/progenitor cells with tumorigenic capacity have been proposed to contribute to tumour heterogeneity [41]. These novel points demonstrate the important traits of MSCs in neoplasia. Furthermore, emerging evidence suggests that MSCs can serve as an attractive target for antitumour therapy [42, 43].

### MSCs: tumour growth

Studies have reported a protumour role of MSCs, and that tumour overgrowth was observed when tumour cells (from lymphoma, melanoma and breast cancer) were coinjected with MSCs into mouse models [23]. Some studies have found that TA-MSCs promote tumour growth in follicular lymphoma, head and neck carcinoma, glioma, breast tumours, gastric tumours, colon tumours and prostate tumours [26, 44–46]. In ovarian tumours, adipose-derived MSCs significantly enhance the growth of SKOV3 tumours in mice, but no obvious growth was observed in the BM-MSC group. Interestingly, a study using luciferase-labelled SKOV3 cells found that the increase in tumour weight was due to the increase in tumour cells and not the expansion of MSCs [47]. Notably, the absolute number of MSCs had no obvious effect on tumour size and had only a modest dose-related effect. In addition, MSCs did not increase angiogenesis, and there was no difference in the number of fibroblastic/stromal cells in tumours in either the BM-MSC group or TA-MSC group [47]. These results clarify that the growth of tumours is independent of expansion and the absolute number of MSCs. MSCs account for a low percentage of cells in tumour tissues, but their potential for inducing tumorigenesis is essential for tumour growth and metastasis. In addition, IL-17, which is an inflammatory factor that contributes to the development and progression of tumours, can be released by various cells, including tumour cells, immune cells and cancer-associated fibroblasts [48–51]. IL-17 promotes the proliferation and migration of TA-MSCs and enhances the stemness of TA-MSCs; it can also activate the NF-κΒ, STAT3 and β-catenin pathways in MSCs, leading to TA-MSC-related autocrine and paracrine signalling that stimulates the proliferation of gastric tumour cells [52]. These results suggest that TA-MSC-mediated tumour growth/proliferation may be associated with autocrine and paracrine signalling.

In contrast, several early studies indicate that MSCs inhibit tumour cell proliferation by secreting various factors [53–55]. In Kaposi’s sarcoma (KS), i.v. injected BM-MSCs inhibit KS tumour growth in vivo by inhibiting Akt activation in a dose-dependent manner; interestingly, this phenomenon was only observed for primary tumour cells but not tumour cell lines [56]. Similarly, Dickkopf 1 (Dkk-1) derived from MSCs -from the human dermis inhibits Wnt/β-catenin signalling in breast cancer cells, leading to a delay in the tumour formation of SCID mice injected with a mixture of tumour cells and MSCs (approximately 20.6 days), while the control group was developed tumours faster (approximately 6.5 days) [54]. However, a study showed that BM-MSCs mixed with weakly metastatic human breast carcinoma cells caused tumour cells to increase their metastatic potency [26]. The reason for this difference is not clear, but it may be related to the number of tumour cells or MSCs used in the experiment and/or associated with the method of injection, tumour type and/or stage and the timing of injection.

### MSCs: tumour metastasis

A study showed that mixed breast tumour cells and BM-MSCs can effectively enhance the capacity of metastasis via CCL5. The group that received MSCs had increased numbers of microscopic and macroscopic lung metastases compared with the negative control, and the metastatic lesions had twofold to sevenfold greater enhancement. These results were only seen when the metastatic lesions were close to the engrafted tumours, and this metastatic phenotype could be reversed by removing the MSCs [26]. As mentioned above, the relationship between tumours and MSCs induces the conversion of MSCs, and the new MSCs promote tumour metastasis. One reason for this is that the fate of disseminated tumour cells (DTCs) influences the metastasis of patients, and DTCs shape the newly active niche by forming premetastatic lesions in distant organs [57]. DTCs may adapt to and alter a pre-existing niche to make it capable of supporting the same rate of proliferation as a metastatic niche. Intriguingly, DTCs can also disseminate to bone marrow to induce harmful effects in prostate and breast tumours [58]. Prostate tumour cells show a high metastatic capacity, and metastasis to secondary sites (such as skeletal tissues) can be enhanced by MSC stimulation via CXCL16/CXCR6 signals [31]. CXCL16 signalling can be induced by IFN-γ, TNF-α and IL-1β, which are involved in the migration and invasion of prostate tumour cells [59]. These results demonstrate that CXCL16 induces not only to the migration of MSCs but also the migration of tumour cells. This effect may be caused by Akt/mTOR signalling [59]. Targeting MSCs and CXCL16/CXCR6 signalling to prevent the tumour recruitment of MSCs may be a novel effective therapeutic strategy for combating tumour metastasis.

## MSCs and the TME

**Fig. 2:**
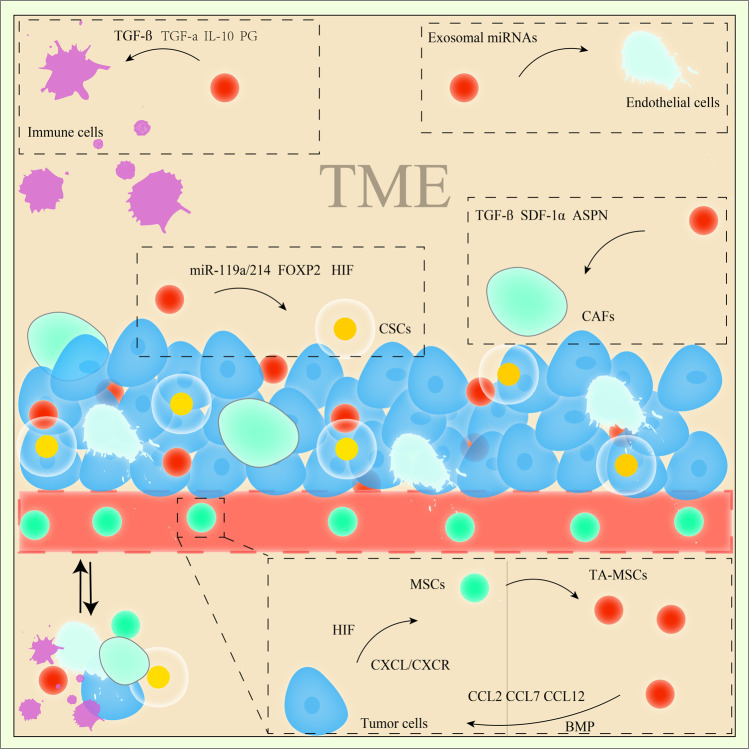
The relationships and mechanism of MSCs in the TME. MSCs can be recruited from other tissues through vessels (while some MSCs reside in the TME) and then transform into TA-MSCs via HIF, CXCL and BMP signalling. TA-MSCs regulate immune cells via paracrine signalling (TGF-α, TGF-β, IL-10, and PG) and transform into CAFs or mediate CAF-related malignant processes via TGF-β, SDF-1α and ASPN signalling. TA-MSCs also maintain the CSC population through miR-119a/214, FOXP2, and HIF signalling.

Paget in 1889 proposed that bidirectional communication between tumours and their surrounding microenvironment plays a central role in tumour proliferation, migration and metastasis, and this idea was named the seed-and-soil hypothesis [24]. Current anticancer therapeutics cannot effectively eliminate all tumour cells in the body, as the ‘soil’ (the tumour stroma) also affects tumours. Some components of the TME (such as endothelial cells (ECs), innate and adaptive immune inflammatory cells and CAFs) are now well recognized to be involved in tumour growth, migration and metastasis [60]. Furthermore, the mesenchymal niche also orchestrates the initiation of tumours and controls tumour-initiating cells (TICs) [61]. Although CAFs are the most abundant cell type in the tumour stroma, some questions related to CAFs remain unanswered, such as whether they originate from endothelial cells or pericytes participating in the formation of new blood vessels. Tumours can be thought of as ‘wounds that never heal’, and there are many kinds of inflammatory cells and factors that exist persistently in the tumour microenvironment [23, 62]. This continuous inflammatory activation in tumours undoubtedly contributes to the migration of MSCs and circulating endothelial precursor cells (CEPs) [60]. These MSCs home to the TME and can be ‘educated’ by the TME to mediate the process of tumour progression [63, 64]. The ‘education’ process in the TME can modulation differentiation and transformation [22, 65]. Intriguingly, in the TME, MSCs can differentiate into adipocytes, endothelial cells and fibroblasts [66, 67]. Various factors, multiple groups of cells and the matrix collectively participate in the complex regulatory mechanism, implying that these factors together drive the development of tumours (Fig. 2).

### Conversion of MSCs into TA-MSCs

MSCs are a class of multipotent cells that are capable of self-renewal and differentiation [68]. MSCs are found in many locations throughout the body and easy to separate, have high expansion rates and high migratory capacity, and can effectively prevent allogeneic responses after transplantation. These unique characteristics have led to extensive application of MSCs in multiple areas.

MSCs can be isolated from various sources, including adipose tissue, periosteum tissue, synovial membrane, synovial fluid (SF), skeletal muscle, testes, deciduous teeth, pericytes, trabecular bone, infrapatellar fat pad, articular cartilage, umbilical cord blood, placenta, liver, spleen, and thymus [69–74]. To date, many studies have found that MSCs can differentiate into adipocytes, chondrocytes, osteocytes, skeletal muscle cells, cardiomyocytes, smooth muscle cells, and other cell types [75, 76]. During the differentiation of MSCs towards a specific cell type, a multitude of physical and chemical factors are involved in the initial commitment and later stages, such as cytokines, growth factors, extracellular matrix factors and transcription factors (TFs) [74]. These variables greatly drive the development of regenerative medicine strategies by researchers.

Recently, many investigations have revealed that the relationship between tumour cells and TA-MSCs promotes tumour growth [26, 46, 77]. MSCs include many subpopulations, and bone marrow MSCs (BM-MSCs) are the most widely studied group involved in tumour development [2]. However, BM-MSCs are different from TA-MSCs. For instance, in ovarian cancer, compared with BM-MSCs from healthy donors, TA-MSCs secrete more molecules, such as bone morphogenetic proteins (BMPs), which are involved in the differentiation of stem cells and the proliferation of tumour cells [78]. Similarly, TA-MSCs were found to produce and secrete more BMP than adipose-derived MSCs [47]. The level of BMP is positively correlated with tumour growth in multiple tumours (such as melanoma and ovarian cancer), and tumour growth can be reversed by BMP antagonists [47, 79, 80]. Conversion of MSCs into TA‑MSCs in multiple tumours induced by the tumour microenvironment changes the characteristics of normal MSCs and promotes the malignant capacities of tumours. Likewise, in melanoma, TA-MSCs release exosomes carrying miRNAs; 16 of these miRNAs were expressed at lower levels while 2 were expressed at higher levels than normal, and the tumour-suppressive miRNA miR-15a was expressed at lower levels than normal [81]. Another study also found that TA-MSCs derived from lymphomas in mice can promote tumour growth, and further studies showed that TA-MSCs can produce high levels of the CC-chemokine receptor 2 (CCR2) ligands CCL2, CCL7 and CCL12, which recruit macrophages into the tumour and lead to the acquisition of an M2­like phenotype [23]. The secretion capacity of TA-MSCs remains even after several passages in vitro [23]. These results indicate that the tumour microenvironment induces permanent transformation of BM­MSCs into TA­MSCs [3, 23, 82, 83]. TA-MSCs from gastric tissue coinjected with gastric tumour cells can enhance tumour growth and angiogenesis; amazingly, tumour cells can also induce the migration, proliferation and differentiation of TA-MSCs into endothelial cells [84]. This MSC-tumour cell communication causes tumour growth, tumour cell migration and angiogenesis. In addition, in mouse models of head and neck carcinoma and gliomas, it has also been proven that TA-MSCs promote tumour [45, 85]. In human ovarian tumours, once the cells are removed from the tumour microenvironment, the gene expression profile of TA-MSCs is maintained for weeks [47]. This phenomenon may hint that an autocrine loop of TA-MSCs is induced by the TME or epigenetic events.

Intriguingly, coculture of L-MSCs with primary BM-MSCs (derived from GFP-transgenic mice) leads to a similar expression pattern of cytokines/chemokines in both L-MSCs and BM-MSCs. This separate type of “MSC” is called BM-MSCs educated by L-MSCs (BM-L-MSCs), and BM-L-MSCs usually function as tumour promotors even after several passages [82]. Another study found that conditioned medium (CM) from tumour cells (MDAMB231, PANC-1 and U87) can activate MSCs from bone marrow in humans, which promotes tumour growth in vivo [65]. The capacity to promote tumour growth implies that some paracrine molecules are involved in this conversion process. Recently, a report also proved that exosomes effectively mediated the conversion [83]. In addition, BM-MSCs treated with tumour necrosis factor (TNF) mimic TA-MSCs, leading to tumorigenesis in mouse models of lymphoma, melanoma and breast carcinoma [23]. These results suggest that MSCs can be ‘educated’ by the tumour microenvironment and evolve into TA-MSCs via cell–cell interactions and paracrine pathways. Although there have been very few studies of the transformation of MSCs into TA-MSCs, these results highlight the necessary role of MSCs in tumour progression.

### Influence of MSCs on immune cells

In 1998, MSCs were first reported to function as immunosuppressive cells [86]. MSCs serve as regulatory factors of tumour immune systems involved in both the innate and adaptive immune systems and shape the microenvironment to mediate tumour progression [23, 87]. The immunosuppressive process involves some factors, including TGF-β, IL-10, indoleamine 2,3-dioxygenase (IDO), prostaglandin (PG) E2 and nitric oxide [88–92]. A study demonstrated that nitric oxide produced by BM-MSCs is an important mediator in the suppression of T cells [88]. TA-MSCs sourced from cervical tumours weaken CD8^+^ T-cell activity to protect tumour cells [93]. In addition, BM-MSCs also play an immunosuppressive role in the development of CD4^+^IL-10^+^ cells (Tr1 cells) and CD4^+^CD25^+^Foxp3^+^ cells (Treg cells) [94, 95]. BM-MSCs secrete TGF-β, which activates Treg cells; these Treg cells have important immunosuppressive effects in breast tumours via CD8^+^ T cells and NK cells [96]. The tumour microenvironment contains not only T cells but also B cells, which promote or inhibit processes to participate in tumour progression [97, 98]. BM-MSCs change the immune response in tumours via production of cytokines and chemokines and can also induce B cells to undergo cell cycle arrest in the G0/G1 phase, inhibiting the proliferation of B cells and antibody production [99, 100]. Furthermore, human adipose-derived MSCs promote Treg cell and CD8^+^ T-cell production of a suppressive phenotype by inducing macrophage-derived TGF-β secretion [101, 102]. In breast tumours, TGF-α is activated in MSCs to recruit CXCR2^+^ neutrophils and promote the metastasis of breast tumours [103]. Moreover, in breast tumours, MSCs can secrete the chemokine CXCL10 and chemokine (C–C motif) ligand 5 to regulate tumour cells. As a feedback loop, tumour cells secrete CXCL16 and CSF1 (colony-stimulating factor 1), both of which depend on the transcriptional activity of HIFs, leading to the recruitment of MSCs and macrophages to primary tumours and mediating the metastasis of tumour cells to lymph nodes and lungs [104]. The above studies demonstrate that MSCs can directly or indirectly mediate immunoregulation by various cytokines and chemokines, including TGF-α, CXCL-10, TGF-β, IL-10, and PG.

Although MSCs play an important role in the immune system, the specific mechanisms of MSCs in the TME are still unclear, and more in-depth explorations of MSCs in solid tumours are needed.

### Regulation between MSCs and CAFs

Numerous studies have noted the prominent functions of CAFs in tumorigenesis and development, which have important clinical implications [105]. The most direct origin of CAFs in the TME is TME fibroblasts and MSCs [22, 106–109]. It is worth mentioning that MSCs can differentiate into fibroblasts in the TME [5]. CAFs are a class of heterogeneous cells in the TME and have unique capacities depending on the tumour characteristics [110]. A study showed that CAFs contribute to the growth initiation and metastasis of tumours [111, 112]. Tumour cells can release multiple factors, such as cytokines, including transforming growth factor-β (TGF-β), which promote the activation of myofibroblasts, leading to extracellular matrix (ECM) synthesis and remodelling during the desmoplastic reaction [113–115]. The ECM interacts with cells to regulate a variety of functions, including proliferation, migration, and differentiation [116]. ECM remodelling is essential for regulating the morphogenesis of organs such as the intestine and lungs [116, 117]. Disorders in the composition, structure, stiffness, and abundance of ECM lead to pathological conditions such as fibrosis and aggressive tumours [116, 118, 119]. A study showed that activation of JNK signalling in metastatic breast tumour cells (metastasis-initiating breast tumour cells) obviously promotes activation of fibroblasts by IL-1α/β, which interact with IL-1R on fibroblasts in the lung to stimulate NF-κB-mediated induction of CXCL-9/10 during the process of lung colonization. The secreted CXCL9 and CXCL10 bind to CXCR3 on the surface of a subpopulation of breast tumour cells, which promotes initiation and growth of metastatic tumour cells [120]. This evidence proves the irreplaceable roles of CAFs in the progression and development of various tumours. The cells from which CAFs originate have been explored, including resident fibroblasts, smooth muscle cells, endothelial cells, epithelial cells, fibrocytes and BM-derived cells [26, 121, 122]. Several studies have reported that MSCs have the capacity to transform into CAFs [26, 31, 84]. MSCs can transform into SMA^+^ MFs, which serve as a subgroup of CAFs and promote a normal BM niche. At least 20% of CAFs originate from BM and are derived from MSCs in gastric tumours. MSC-derived CAFs can be recruited to the dysplastic stomach in a TGF-β- and SDF-1α-dependent manner and express IL-6, Wnt5α, and BMP4 and have DNA hypomethylation, leading to tumour growth [22]. In prostate tumours, inhibiting CXCR-6 expression can interrupt the differentiation of MSCs into CAFs and decrease the CXCL-12 secretion of CAFs via Erk and NF-κB signalling, and knockdown of CXCL16 in vitro also inhibits the formation of CAFs [31]. The small leucine-rich proteoglycan (SLRP)-secreted protein asporin (ASPN) is obviously enriched during the development of articular cartilage [123, 124]. ASPN binds to BMP-4, inhibiting the differentiation of MSCs into CAFs and leading to the migration of tumour cells and MSCs [5]. Some CAFs originate from MSCs, and research has shown that CAF-MSCs (CAFs with MSC characteristics) from neuroblastoma (NB) enhance tumour engraftment, growth and resistance to etoposide or melphalan via STAT3 and ERK1/2 signalling in vivo [125]. The abovementioned results show that in the TME, MSCs are involved in CAF regulation, and MSC-like features mediate the link between CAFs and tumour cells.

The transformation of MSCs into CAFs involves many molecules, and an interesting study found that the stiffness of collagen-coated polyacrylamide gels affects differentiation. Compared with those grown on softer gels, MSCs grown on stiffer polyacrylamide gels had enhanced expression of YAP in the nucleus and exhibited a spread morphology and higher expression of CAF markers, suggesting that the differentiation of MSCs into CAFs depends on the activation of YAP-mediated mechano-signal transduction [126]. Biomolecules and chemicals in the TME commonly serve as important factors in tumorigenesis and development; furthermore, physical factors (such as mechano-signal transduction) also contribute to maintaining the integrity of the TME and promoting the differentiation of MSCs into CAFs.

Although many studies have clarified the significance of MSCs and CAFs, some questions remain unanswered: what percentage of MSCs in the TME differentiate into CAFs in different tumour types? What is the main mechanism that regulates the conversion of MSCs into CAFs? How do CAFs regulate MSCs?

### Relationship between MSCs and endothelial cells

New blood vessel formation is essential for tissue repair, inflammation and tumour progression and metastasis. Once tumours grow beyond a few millimetres, they require a mass of endothelial cells to build more vessels. The formation of tumour microvessels is a challenge in the process of tumour growth and metastasis. Evidence suggests that MSCs tend to reside near the perivascular niche, which is in close proximity to endothelial cells (ECs) [127]. Gastric tumours contain many MSCs and have high vascularity; importantly, tumour cells can enhance the endothelial differentiation capacity of tumour-resident MSCs [84]. Although the specific mechanism of differentiation is still unclear, mediators are considered to be involved in the transfer of information. Exosomes are a class of small membrane vesicles carrying abundant mRNAs, microRNAs (miRNAs), small molecules and proteins that can be released constitutively via multivesicular body (MVB) fusion with the cell membrane [128, 129]. Groups of exosomal RNAs or proteins shuttled between donor cells and recipient cells transmit functional signalling. Exosomes from stage IV melanoma have a higher exosome protein concentration than those from melanoma of other stages and normal controls. In individuals with the same stage of disease, those with protein-poor exosomes (<50 μg/ml) had better survival, 4.5-times higher recruitment of tumour-derived exosomes by MSCs, and 3-times higher vascular density than those with protein-rich exosomes (>50 μg/ml) [130]. Exosomes may be hubs of bidirectional communication.

### Effect of MSCs on CSCs

The origin of tumour cells remains unclear. On the one hand, a study proved that tumours can arise from cells with abnormal differentiation [131]. On the other hand, intriguingly, CSCs, a rare population with stem cell properties in tumour tissues, have been proposed to mediate the initiation of tumours and repopulate tumour cells [132, 133]. For example, in human melanoma, only 0.0001% of melanoma cells are tumorigenic [134]. Indeed, the vast majority of tumours have only rare (<0.1%) CSCs in tissues in mouse models [135]. CSCs are a small minority population in the TME, and developing anticancer strategies to kill these CSCs rather than the bulk populations of tumour cells will improve therapy [136]. Tumour cells serve as abnormal stem/progenitor cells in tumour tissues and are highly related to drug resistance, poor survival and tumour recurrence [38]. CSCs usually account for a small percentage of tumour cells. However, in the process of tumorigenesis, when a critical mass of CSCs is achieved, the CSCs then undergo differentiation as opposed to self-renewal. These properties may explain why CSCs have substantial effects despite their lower abundance in tumours.

It has been proposed that tumour cells do not harbour tumorigenic potential and mainly differentiate from CSCs [137]. Furthermore, whether so-called CSC multipotency and asymmetric division are present in solid tumours remains unclear. Consequently, the alternate term “tumour-initiating cells” was suggested to describe the characteristics of the specific population of tumour cells [138]. CSCs, similar to normal stem cells, have self-renewal and differentiation capacities. In 1978, Schofield proposed the concept of a stem cell niche, a physiological microenvironment that maintains the stemness of stem cells [139]. Researchers have found that MSCs can maintain the CSC niche to mediate tumour progression. TA-MSCs increase the number of CSCs via BMP signals, which indicates that MSCs can promote the self-renewal of CSCs in ovarian tumours, with a 4-8-fold increase in the percentage of CSCs compared with BM-MSCs in vivo or in vitro [47]. In breast tumours experiments using ALDEFLUOR and DsRed-labelled SUM159 cells, coculture with MSCs resulted in an approximately 14% increase in ALDEFLUOR-positive cells, which suggested that MSCs promote the self-renewal of CSCs and that CXCL1, CXCL5, CXCL6, IL-6 and IL-8 are involved in this process [140]. Similar studies have clarified the key role of cytokines and chemokines in MSC-mediated malignant effects [31]. Not only these factors but also hypoxia (1% O_2_) and HIFs induce effects on the normal TME [33]. Cytokines and chemokines produced by paracrine signals are a result of MSCs, and cytokines and chemokines can transmit information between MSCs and CSCs. Interestingly, some microRNAs promote the induction of CSC-like phenotypes, leading to tumour metastasis [141]. MicroRNAs (miRNAs, or miRs) are a class of noncoding RNAs that can regulate gene expression mainly by combining with mRNA-specific sequences, leading to the translational inhibition or degradation of target sequences [142]. TA-MSCs enhance the stemness of breast tumour cells, resulting in anoikis resistance, tumour initiation, metastasis and relapse via cell–cell interactions. These effects promote Twist-mediated miR-199a/214 transcription, and miR-199a/214 increased the accumulation of stem cell-associated factors by inhibiting FOXP2 (transcription factor forkhead-Box P2) expression [141]. Coding proteins, noncoding RNAs, cytokines, chemokines and even stressors in the microenvironment all participate in MSC regulatory networks, mediating bidirectional communication of MSCs and CSCs. These factors may be able to be targeted to prevent tumour metastasis or relapse. The clinical medicine digoxin can inhibit HIF activity. After 1 week of digoxin treatment in vitro, tumour recruitment of BM-MSCs was interrupted, decreasing lymphatic metastasis and lung metastasis [33].

Combining digoxin with chemotherapy to cure solid tumours that possess high metastatic capacity or are prone to relapse may be a feasible approach. In addition to pharmacological strategies, inhibiting CSC self-renewal and interrupting the bidirectional communication between CSCs and other subpopulations of cells may be novel ways to cure solid tumours. Physically sequestering MSCs helps disrupt the niche, leading to altered bidirectional communication between MSCs and CSCs to achieve growth inhibition. It is likely that targeting unique combinations of pharmacological mechanisms and/or interactions for different tumour types will lead to the most efficient therapeutic effect.

## MSCs and oncotherapy

At present, many strategies, including chemotherapies, targeted therapies, radiotherapies and immunotherapies, can lead to tumour shrinkage, inhibition of metastasis and even tumour cure. However, resistance to oncotherapy can develop in multiple ways. Tumour cells as well as stomal cells, including TA-MSCs, shape a protumour microenvironment to promote tumour growth and metastasis in patients undergoing chemotherapy and radiotherapy [143, 144]. MSCs play an important role in tumorigenesis and drug resistance and are involved in various pathways. We have summarized the content as follows.

Some in vivo studies have found that TA­MSCs or BM­MSCs can induce tumour cell resistance to oncotherapy [145]. A study showed that endogenous mesenchymal stem cells (MSCs) can release various factors during treatment with platinum analogues, such as polyunsaturated fatty acids (PIFAs), 12-oxo-5,8,10-heptadecatrienoic acid (KHT) and hexadeca-4,7,10,13-tetraenoic acid (16:4(n-3)), which protect tumour cells against a range of chemotherapeutics (cisplatin and oxaliplatin) and block central enzymes to inhibit PIFAs and prevent MSC-mediated resistance [146]. Notably, MSCs from bone marrow can release fatty acids to decrease drug efficiency. Moreover, NO, as a soluble factor produced by TA-MSCs, has been shown to enhance etoposide resistance in pancreatic tumours in vitro, and IL-1β from tumour cells is an effective stimulator that increases the release of NO [147]. Interestingly, IL-1β and NO can form a positive feedback loop: tumour cells and immune cells produce IL-1β, leading to the release of NO. NO in turn induces the secretion of IL-1β, thus becoming further enhancing resistance to chemotherapy (etoposide) [147]. In addition, in head and neck squamous cell carcinoma cells, BM-MSCs mediate the resistance to paclitaxel in vitro according to transwell culture experiments [148]. In ovarian cancer, BM-MSCs produce CXCL-12 to promote thermotolerance, which can protect tumour cells against chemotherapy [149]. These results suggest that TA-MSCs can provide resistance for tumour cells, leading to unsatisfactory clinical treatment. BM-MSCs can be recruited to prostate tumours when castration induces inflammatory activation, which accelerates hormone resistance via chemokine ligand 5 secretion [150]. These results provide a novel idea: blocking MSC migration may suppress castration resistance in prostate tumours. Although direct depletion of TA­MSCs is difficult in the TME, because they express a limited set of cell surface markers, upstream and downstream modulators of TA­MSCs during tumorigenesis and development have been gradually clarified, and it is possible to cure tumours by targeting these factors with new anticancer strategies.

## Perspectives: MSC exosomes

MSCs have demonstrated great potential in tumour therapies. However, the specific mechanism by which cell–cell communication is mediated is uncertain. The mechanisms by which MSCs and other cells transform and induce signal transduction also remain to be revealed. An increased understanding of the development of exosomes, which are extracellular vesicles (EVs) with a diameter range of 40–100 nm, and cell communication will help answer these questions. In response to cell-to-cell contact, MSCs can produce abundant exosomes and thus play a key role in mediating tumour progression [151, 152]. Exosomes from MSCs are ideal carriers because they have a considerable ex vivo expansion capacity, tropism for tumour tissue, and low immunogenicity and induce few side effects [19, 81, 153, 154]. Nanodrugs (doxorubicin) loaded into exosomes have been shown to cure osteosarcoma and induce excellent effects: this strategy enhanced the cellular uptake efficiency and antitumor effect but had low cytotoxicity in myocardial cells [155]. In addition, exosomes derived from MSCs can also contribute to immunoregulation and inflammation. Exosomes derived from MSCs can ameliorate liver fibrosis by inhibiting both the epithelial–mesenchymal transition and collagen production, and they also inhibit inflammation [156]. For liver tumours, especially liver fibrosis-related tumours, MSCs may be a perfect target to cure tumours.

On the other hand, the indirect effects of EVs can lead to tumour progression. MSC-derived EVs induce different effects in different tumours in in vivo assays of tumour cell apoptosis and proliferation and tumour formation [157]. These findings substantially improve our knowledge, and direct or indirect effects of MSCs identified in patients or tumour models play an important role in tumorigenesis, tumour progression and even oncotherapy. Physical signals, chemical molecules and biomolecules that crosstalk with and activate each other constantly shape a niche for tumour survival. Interruption of cell information transduction in the TME in specific ways may be a useful strategy for curing not only cancers but also some ageing-related diseases and for regenerative medicine. In the future, such strategies may help realize a world without disease.

## Conclusions

Every higher organism has billions of cells that make up different tissues and organs. Given that genetic information is the same in all cells, including the stem cells, cells of higher organisms are not told how to function on their own. Therefore, for each organ to operate successfully within the context of the organism, all cells must be integrated into both architectural and signalling frameworks such that each cell knows exactly which commands to execute at any given time. Homoeostasis is achieved when these frameworks are successful, while their failure results in a spectrum of dysfunctions, including cancer. How do organisms achieve this remarkable feat, and how does each cell know what to do within the tissues? Cell-to-cell interactions, including material transport and information exchange, are vital to these processes. MSCs possess some unique characteristics, such as tumour tropism and their capacity for differentiation and self-renewal. Although modifying MSCs in the TME is difficult, an increased understanding of upstream and downstream relationships will inform strategies that take advantage of the characteristics of MSCs to inhibit tumour growth and metastasis in the future.

1. Inhibiting the secretion of chemokines that are required for the recruitment of MSCs to tumours.
2. Decreasing TA­MSC-produced growth factors that can promote angiogenesis as well as the survival and proliferation of tumour cells.
3. Suppressing molecules or factors in combination with oncotherapy to inhibit tumour cell resistance to chemotherapy and radiotherapy.
4. Genetically modifying MSCs or their exosomes and using the tropism of MSCs to target and kill tumour cells.

Although many studies are in the preclinical stages, some strategies are being tested in clinical settings. MSCs engineered to express IFNβ or thymidine kinase, which is an enzyme capable of converting the prodrug ganciclovir into ganciclovir triphosphate, are now being examined for their therapeutic effects in patients with ovarian cancer and gastrointestinal cancer (Clinicaltrials.gov UNLoM. https://www.clinicaltrials.gov/ct2/show/NCT02068794?term=NCT02068794&rank=1; https://www.clinicaltrials.gov/ct2/show/NCT02530047?term=NCT02530047&rank=1; https://www.clinicaltrials.gov/ct2/show/NCT02008539?term=NCT02008539&rank=1). In the future, targeting stromal stemness may be a novel strategy to improve clinical treatment.

## Supplementary information

Editing Certificate
