# Supplementary material for: Mesenchymal/stromal stem cells: necessary factors in tumour progression
Source: Cell Death Discov. 2022 Jul 22;8:333. doi: 10.1038/s41420-022-01107-0 (PMC9307857; doi:10.1038/s41420-022-01107-0)
Supplement: Supplementary file 1 — Editing Certificate [file 41420_2022_1107_MOESM1_ESM.pdf]

SPRINGER NATURE

Author Services

Editing Certificate

This document certifies that the manuscript

Mesenchymal/ stromal stem cells: inseparable actors in tumor progression

prepared by the authors

Xinyu Li, Qing Fan, Xueqiang Peng, Shuo Yang, Shibo Wei, Jingang Liu, Liang Yang, Hangyu Li

was edited for proper English language, grammar, punctuation, spelling, and overall style by one or more of the highly qualified native English speaking editors at SNAS.

This certificate was issued on **June 5, 2022** and may be verified on the SNAS website using the verification code **70AD-6352-FF7D-B755-26FB** .

Neither the research content nor the authors' intentions were altered in any way during the editing process. Documents receiving this certification should be English-ready for publication; however, the author has the ability to accept or reject our suggestions and changes. To verify the final SNAS edited version, please visit our verification page at secure.authorservices.springernature.com/certificate/verify.
If you have any questions or concerns about this edited document, please contact SNAS at support@as.springernature.com.

SNAS provides a range of editing, translation, and manuscript services for researchers and publishers around the world.
For more information about our company, services, and partner discounts, please visit authorservices.springernature.com.
